# Thirteen New Cases of Tuberculosis in the Dog

**Published:** 1896-07

**Authors:** 


### ABSTRACTS FROM FOREIGN JOURNALS.

UNDER THE DIRECTION OF

J. PRESTON HOSKINS, PRINCETON, N. J.

THIRTEEN NEW CASES OF TUBERCULOSIS IN THE DOG. The dog was formerly considered to be proof against tuberculosis, but since autopsies are carried out with greater care, and especially since we possess in the bacillus of Koch a sure means of diagnosis, this opinion has been proven erroneous. There have been recently published abroad at least 100 cases of alleged tuberculosis in the dog. Cadiot alone has reported more than 40, Jensen 28, Eber 11, and Fröhner, who observed 27 last year, has just announced a series of 13 more. The Berlin professor takes advantage of the occasion to make some general remarks upon the chemical, anatomical, and pathological characteristics of tuberculosis in the dog, and gives in detail his observations. We shall confine ourselves to the general remarks. Almost always suspicion as to the existence of the disease is first aroused on hearing the statements relative to the subjects submitted for examination. In most cases it is learned that the dog coughs or has been short of breath for some time; that he has been running down; and that all remedies employed have proved ineffectual. In some cases the disease has developed very rapidly—in two or three weeks; at other times the cough and difficulty in breathing are lacking, but there exist a serious loss of appetite and very rapid emaciation. In the majority of cases tuberculosis presents the clinical symptoms of a chronic affection of the lungs or of the pleura; more rarely it shows itself in the form of a chronic catarrh of the bronchus, or of the hydrothorax. Progressive emaciation combined with the existence of an irregularly intermittent fever strengthen the suspicion. In seven cases Fröhner made use of tuberculin in the form of a hypodermic injection; five times the temperature rose from 0.8° to 1.5°, once the elevation was only 0.4°, and in the last case the thermometer showed a fall of 0.5°. In some cases the reaction followed after two, three, or five hours; again it did not appear until ten or twelve hours had passed.

The lesions found at the autopsy varied according to the age and the localization of the disease, the organs of the thorax being those most frequently affected. In the last thirteen cases observed by the author the lesions occupied the lungs and pleura 11 times, the liver 10 times, the bronchi and the ganglions of the mediastinum 6 times, the pericardium 5 times, the kidneys 3 times, the spleen 1 time, all organs involved 1 time. In the lungs Fröhner has found oftenest the cavities and the caseous masses extending as far as the pleura. In some cases the lungs presented large pneumonic nodosities or small miliary granulations of a tuberculous nature. Often there was adhesion of the lung to the costal wall; sometimes this adherence extended to the neighboring ganglions; accompanying the lesions of the lungs atelectasis, œdema, emphysema, and bronchiectasis were noted. The pleural lesions consist sometimes of a serous or serofibrinous pleurisy, more rarely hemorrhagic, with considerable effusion (overflow); sometimes of a dry, granular, and adhesive pleurisy. At times the author observed disseminated miliary tubercles upon the pleura accompanying empyema or hydrothorax. The inferior cervical and the anterior and posterior mediastinal bronchial ganglions were enlarged and often adhered to one another, forming sometimes a mass as large as the fist, which upon being cut showed a pale-gray color and either soft cavities or tubercular granulations. The mediastinum was almost always much thickened. The pericardial lesions were sero-sanguinous effusion, fibro-granulous and adhesive inflammation of the serous membranes, and miliary tubercular infiltration of the walls of the sac. The liver, spleen, and kidneys presented miliary granulations.—*Monatsheft für prakt. Thierheilkunde*, vol. vi. Heft 9, from *Annales de Médicine Vétérinaire*.

